# Effects of High-CO_2_ Fumigation on *Sitophilus granarius* (L.) Life Stages in Wheat Under Different Temperatures and Exposure Durations

**DOI:** 10.3390/insects17090903

**Published:** 2026-08-28

**Authors:** Ammara Asghar, Ilaria D’Isita, Danial Fatchurrahman, Onofrio Marco Pistillo, Maria Luisa Amodio, Raffaele Simonelli, Giancarlo Colelli, Giacinto Salvatore Germinara

**Affiliations:** 1 Department of Agricultural Sciences, Food, Natural Resources and Engineering (DAFNE), University of Foggia, Via Napoli 25, 71122 Foggia, Italy; ammara.asghar@unifg.it (A.A.); giancarlo.colelli@unifg.it (G.C.);; 2 SAIM Impianti S.r.l., 04022 Fondi, Italy

**Keywords:** stored wheat, granary weevil, alternative control methods, modified atmosphere, carbon dioxide fumigation

## Abstract

*Sitophilus granarius* (L.) is a major primary pest of stored grain worldwide. This study evaluated the efficacy of 100% CO_2_ against adults and immature stages (eggs, larvae, and pupae) of *S. granarius* at 25 °C (optimal temperature) and 37 °C (sublethal thermal stress) after three, six, 12, and 21 days of exposure. Both CO_2_ and temperature significantly affected insect survival, although susceptibility, evaluated as the suppression of adult emergence for immature stages, differed among life stages. While treatment with 100% CO_2_ achieved complete adult mortality within three days at both temperatures, exposure to 37 °C alone induced 100% mortality within 12 days. Eggs were the most susceptible immature stage: 100% CO_2_ completely prevented adult emergence after 12 days at 25 °C and three days at 37 °C, while exposure to 37 °C alone was sufficient to inhibit emergence. In contrast, neither larval- nor pupal-infested wheat showed complete suppression of adult emergence following CO_2_ treatment. At 25 °C, CO_2_ significantly reduced adult emergence from both stages at all exposure durations, whereas at 37 °C significant reductions were detected only after three days, as thermal stress alone had already markedly reduced emergence. These findings demonstrate that exposure to 100% CO_2_ alongside sublethal thermal stress provides an overall increase in mortality across life stages, although larvae and pupae remain substantially more tolerant than adults and eggs.

## Simple Summary

*Sitophilus granarius* is one of the most destructive insect pests of stored wheat, causing substantial economic losses worldwide. The use of carbon dioxide (CO_2_) as an alternative to conventional chemical fumigants is attracting increasing interest because it leaves no harmful residues and may offer a more sustainable approach to pest management. In this study, we evaluated the effectiveness of 100% CO_2_ against adults, eggs, larvae, and pupae of *S. granarius* at a normal storage temperature (25 °C) and under moderate heat stress (37 °C). Adults and eggs were highly susceptible to the treatments, whereas larvae and pupae were more tolerant. Exposure to 37 °C was enough to induce complete adult mortality after 12-day exposure and to inhibit the adult emergence from egg-infested wheat. Combining heat stress with elevated CO_2_ concentration reduced adult emergence from larvae and pupae infested in wheat. These results show that the success of CO_2_ fumigation depends strongly on the insect developmental stage and highlight the need to optimize treatment conditions to ensure effective control of the most tolerant stages. The findings provide useful information for developing safer and more sustainable strategies for protecting stored grain.

## 1. Introduction

Postharvest losses of cereal grains during storage represent a major threat to global food security. In developing and grain-exporting countries alike, such losses can severely impact supply chains, reduce market value, and threaten the availability of safe, high-quality food for both domestic consumption and international trade [1]. Insect infestations are responsible for significant quantitative and qualitative damages, causing changes in grains’ nutritional value and altered chemical composition [2]. Insect pests also introduce contaminants such as silk threads, body fragments, hastisetae, excreta, and chemical secretions. Furthermore, insect activity in stored products increases the moisture content favoring the growth of molds that produce mycotoxins [2].

*Sitophilus* species have been reported to cause 10–30% losses to stored cereals in general worldwide, with up to 50% or more under improper storage environments [3]. In particular, *Sitophilus granarius* (L.) (Coleoptera, Curculionidae), commonly known as the granary weevil, has gained economic importance all over the world causing severe damages of stored cereals every year [4]. It is known to be a leading primary pest of wheat, barley, maize, and other grains in storage [5] where the intensity of its damage on stored grains differs, and is associated with cultivar-specific properties of wheat [6,7,8]. The granary weevil females bore through individual grain kernels and deposit a single egg inside, sealing the cavity before moving on, and all subsequent juvenile stages including eggs, larvae, and pupae develop entirely within the kernel [9]. The endophytic development of immature stages makes this species particularly challenging from a pest management perspective causing hidden infestations difficult to detect [10].

Minimizing postharvest losses caused by insect pests, including *S. granarius*, is critical not only for economic sustainability but also for global food security and safety, particularly in regions where wheat is a dietary staple and a primary source of calories and protein [2]. Implementing effective and sustainable pest control measures is therefore essential to maintaining food quality, reducing waste, and supporting long-term agricultural resilience. On the industrial scale, chemicals are being commonly used to control storage pests in cereal grains, although recently more interest has been directed towards non-chemical methods [11]. Indeed, chemical pesticides come with the risk of inducing acute and chronic impacts on human health and adverse effects on environment as well [12]. Thus, mainly in developed countries, legislation limits on the use of pesticides are increasing [13]. For instance, methyl bromide, the primary chemical fumigant historically used against *S. granarius* and other stored-grain pests, has been progressively phased out under the Montreal Protocol due to its ozone-depleting properties. Phosphine became the main replacement fumigant, but resistance to it has now become widespread in stored-product Coleoptera across the globe [14]. This situation has created an actual need for alternative low-impact control tools that are compatible with food safety requirements and grain quality standards [15,16,17].

An alternative to chemical pesticides is the use of modified atmospheres (MAs) that could play an important role in integrated pest management strategies as physical control methods [18]. MA is a low-impact alternative control mean, which can provide disinfestation of stored products from a wide range of pests without leaving toxic residues [19]. The application of MA results in the change in the concentration of gases like carbon dioxide (CO_2_), oxygen (O_2_) and nitrogen (N_2_) in the stored product environment, which results in an atmosphere that is toxic for the target species [20]. Among the options being explored, high-CO_2_ fumigation has received considerable research attention in recent years. Carbon dioxide is residue-free and works by exploiting the respiratory physiology of insects to create lethal anoxic or hypercapnic conditions that insects simply cannot survive with [19]. The effectiveness of CO_2_ fumigation against stored-grain insects depends heavily on several interacting factors, mainly exposure duration, treatment temperature, and which life stage is being targeted [21]. Like all holometabolous insects, *S. granarius* shows considerable variation in susceptibility to fumigants across its life cycle [22]. Immature stages are generally more tolerant than adults, and this is thought to be related to their lower metabolic rates, reduced respiratory activity, and the additional physical protection provided by the grain kernel itself [21]. Within this framework, temperature plays a critical regulatory role. The thermal limits of insects and mites usually fall between 0 and 45 °C, and temperatures within these limits determine the rates of population growth [11]. Conventional thermal disinfestation requires elevated temperatures generally in a range of 45–55 °C to induce control insect infestations. However, the successful application of these treatments relies on a delicate balance between commodity tolerance and insect intolerance [23]. For *S. granarius*, temperatures from 35 °C and above represent an upper marginal threshold that exceeds the species’ limit for normal development [24]. Furthermore, exposure to this thermal range induces in insects a sublethal stress condition characterized by accelerated metabolism and increased respiratory turnover [23]. The increased spiracular opening and gas exchange are expected to act in combination with elevated CO_2_ levels, potentially enhancing the lethal effects of the fumigant. However, whether this interaction remains synergistic, additive, or even antagonistic across different life stages remains inconsistent in the literature [19,25].

In this context, the present study aimed to evaluate the survival of *S. granarius* adults, eggs, larvae, and pupae exposed to 100% CO_2_ fumigation for three, six, 12, and 21 days. Furthermore, to investigate the potential interaction between gas efficacy and temperature, bioassays were conducted at 25 °C, representing optimal developmental conditions, and 37 °C, serving as a short-term sublethal thermal stress environment. To isolate the specific effect of CO_2_ from that of thermal stress alone, control treatments were performed at both temperatures using atmospheric air (0.03% CO_2_).

## 2. Material and Methods

### 2.1. Insects

*Sitophilus granarius* adults were collected in 2022 from wheat storage facilities of the Apulia region in Italy and reared in the dark at 25 ± 2 °C and 60 ± 5% relative humidity (RH) for several generations on durum wheat kernels (var. Simeto) in cylindrical glass containers (Ø 15 × 15 cm) covered with a fine mesh net (0.5 mm) to ensure ventilation while preventing escape.

For the experiments, adult weevils from the eighth generation were obtained directly by sieving grains from the established rearing. To obtain the immature stages, 1000 adult (3–4-week-old) beetles of mixed sexes were first introduced onto 1 kg of uninfested grains (var. Brigante) and maintained for one week in laboratory-controlled conditions (dark, 25 ± 2 °C, 60 ± 5% RH), after which they were removed [11]. Three different sets of infested wheat were prepared to obtain eggs, larvae and pupae. For the egg stage (0–7 days old), infested grain was used as bioassays immediately after the removal of the adults. To obtain the larval stage, the infested grain was incubated further for two weeks ensuring the predominant presence of mid-to-late instar larvae (14–21 days old) in the kernels [11]. To confirm the presence and developmental stage of internal larvae, representative subsamples of kernels were randomly selected and dissected under a stereomicroscope prior to treatment. For the pupal stage the infested grain was monitored until the first adult emergence was observed. At this point, subsamples of kernels were dissected under a stereomicroscope to verify that the remaining internal population consisted predominantly of pupae before initiating the exposure.

### 2.2. Bioassays

Bioassays were conducted using a full factorial experimental design to evaluate the effects of CO_2_, temperature and exposure duration on different *S. granarius* life stages. For treatment on the adult stage, cylindrical plastic containers (Ø 6 × 8 cm), covered with screw caps with a central hole (2 cm) screened by a metallic net (mesh size of 1 mm), were filled with 100 g of durum wheat (*cv* Brigante, 13.5% ± 0.5% w.b. moisture content). After that, wheat samples were infested with a set number (*n* = 20) of *S. granarius*. In the case of immature stages, containers similar to those described above were filled using wheat infested by eggs, larvae, or pupae. Then, wheat samples infested by each stage were maintained at 25 or 37 °C in atmospheric air (0.03% CO_2_) or with 100% of CO_2_. Exposure durations were set to 3, 6, and 12 days for adults, and to 3, 6, 12, and 21 days for immature stages (eggs, larvae, and pupae). Proper additional sets of untreated infested grains were used as controls.

After treatment, an adult was classified as dead if it was immobile and non-responsive to gentle mechanical stimulation. For eggs, larvae, and pupae, the primary endpoint was the total number of adults that subsequently emerged from the treated wheat kernels over the post-treatment monitoring period. New adult emergence was monitored at weekly intervals until no adults emerged for five consecutive days [26].

### 2.3. CO_2_ Fumigation Treatment

Fumigation was carried out using food-grade CO_2_ supplied from a gas management system located in the Laboratory of Research Unit on Postharvest Technology (Department DAFNE, University of Foggia, Italy). Cylindrical plastic containers filled with wheat infested by adults or immature stages as described above were used as sub-replicates. Thus, two containers were placed inside hermetically sealed glass jars. For each stage, three biological replicates, each comprising two sub-replicates (six sub-replicates in total per treatment group per time point), were established for each treatment combination. The CO_2_ was maintained at 100% *v*/*v* throughout the treatment period by continuous purging. The CO_2_ concentration was verified daily using a Dansensor CheckPoint 3 gas analyzer (Ametek Mocon, Ringsted, Denmark), with measurements taken at the gas inlet to confirm that the target concentration was being delivered throughout the exposure period. Temperature treatments of 25 °C and 37 °C (±0.5 °C) were maintained and humidity-controlled environmental chambers were set at 65 ± 5% RH. Exposure was terminated at 3, 6, 12, and 21 days (3, 6, and 12 days for adults), at which point the grain was removed and mortality or adult emergence was assessed.

### 2.4. Statistical Analysis

Data of experiments were analyzed separately for each life stage (adults, larvae, pupae, eggs) using Generalized Linear Mixed Models (GLMMs) fitted in SPSS (Statistical Package for the Social Sciences) v.18 for Windows (SPSS Inc., Chicago, IL, USA). The effects of temperature (25 °C, 37 °C), CO_2_ concentration, and time of exposure (3, 6, 12, and 21 days) were included as fixed factors, along with three-way interactions between them, with replicate membership as a random effect. For treatments on *S. granarius* adults, the GLMM with a zero-inflated Poisson distribution was performed to test for the effect of above-mentioned factors on the number of dead insects, while the GLMM with a negative binomial distribution was performed to test for the effect of the same factors and their interactions on adults emerged from treated wheat infested by eggs, larvae, and pupae. Post hoc analyses with Bonferroni correction (*p* < 0.05) were performed using the estimated marginal means to test the effect of each variable.

## 3. Results

### 3.1. Adult Mortality

According to the GLMM, the mortality of *S. granarius* adults was highly significantly influenced by temperature, CO_2_ concentration, time of exposure, and their three-way interaction (Table 1).

**Figure 1:**
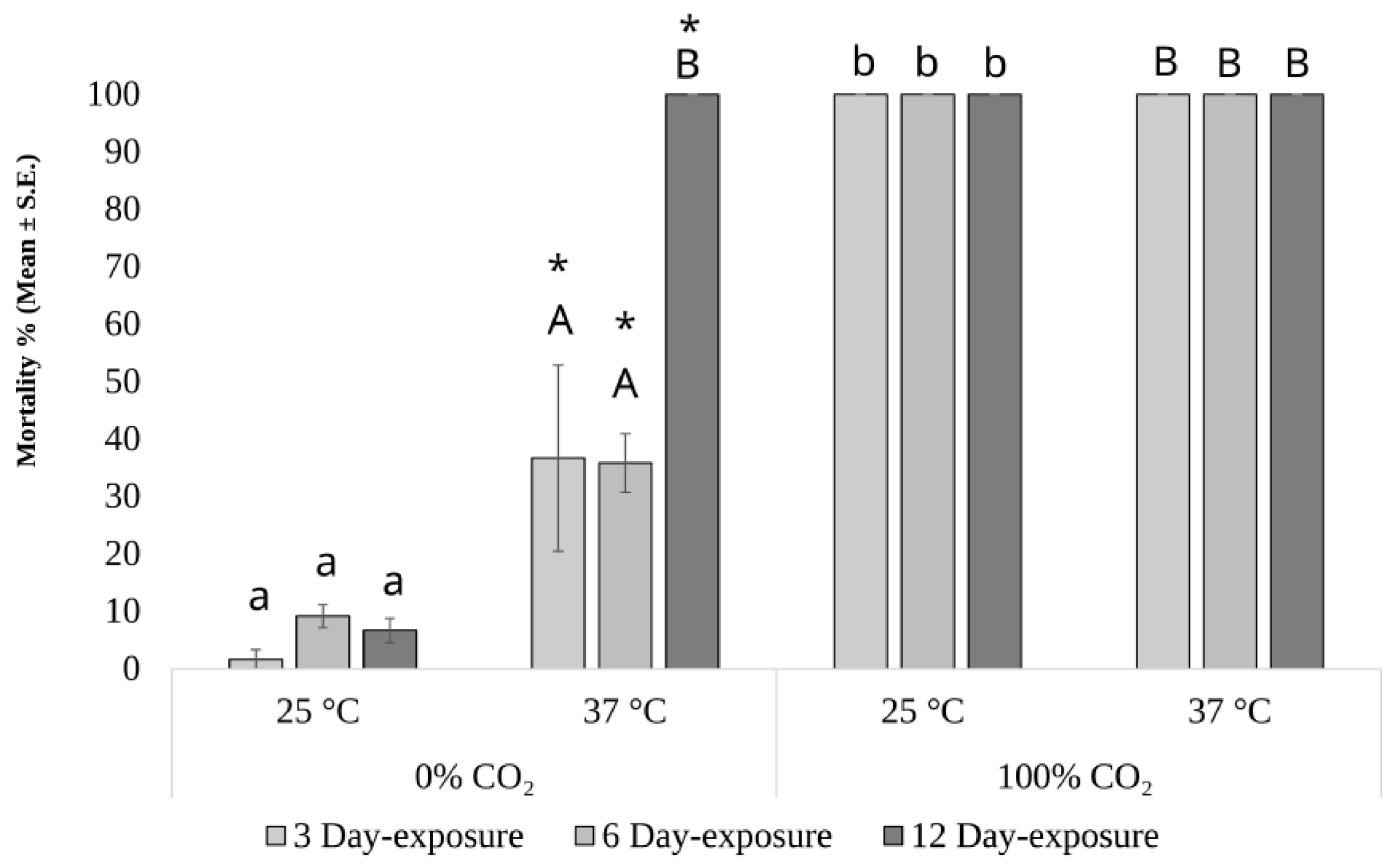
Mortality percentage (Mean ± S.E.) of *S. granarius* adults after 3, 6, and 12 days of exposure to 0 and 100% of CO_2_ and maintained at 25 or 37 °C (N = 3 biological replicates per treatment group, each containing *n* = 20 adults). Different letters indicate significant differences (GLMM post hoc Bonferroni corrected, *p* < 0.05) between CO_2_ doses within the same temperature and exposure day. Asterisks indicate a statistically significant (GLMM post hoc Bonferroni corrected, *p* < 0.05) effect of temperature when comparing the 37 °C thermal condition against the respective 25 °C at the same dose and exposure time.

In the control mortality remained lower than 10% regardless of the duration of the experiment (Figure 1). The sole exposure to 37 °C resulted in a significantly (*p* < 0.001) higher mortality compared to the 25 °C control across all sampling times (Figure 1). In particular, within the 37 °C non-fumigated treatment, insect mortality showed a significant (*p* < 0.001) time-dependent increase, remained constant between day 3 and day 6, and reached complete (100%) mortality after 12 days, indicating that thermal stress alone becomes fully lethal under prolonged exposure. Under 100% of CO_2_, the total adult mortality was achieved by 3 days at both 25 and 37 °C, showing no significant differences over time. In particular, the mortality reached at 25 °C under 100% of CO_2_ was significantly (*p* < 0.001) higher compared to the untreated control for all times of exposure (Figure 1). At 37 °C, the CO_2_ treatment also caused a significantly (*p* < 0.001) higher mortality than non-fumigated treatment at day 3 and day 6. However, by 12 days of exposure, the difference between the two CO_2_ concentrations at 37 °C was no longer significant, as the non-fumigated insects had also reached 100% mortality (Figure 1).

### 3.2. Adult Emergence from Wheat Kernels Infested by Eggs, Larvae and Pupae

The GLMM analysis revealed that adult emergence from treated eggs, larvae, and pupae was highly significantly affected by temperature, CO_2_ concentration, exposure days and their three-way interaction (Table 1).

**Figure 2:**
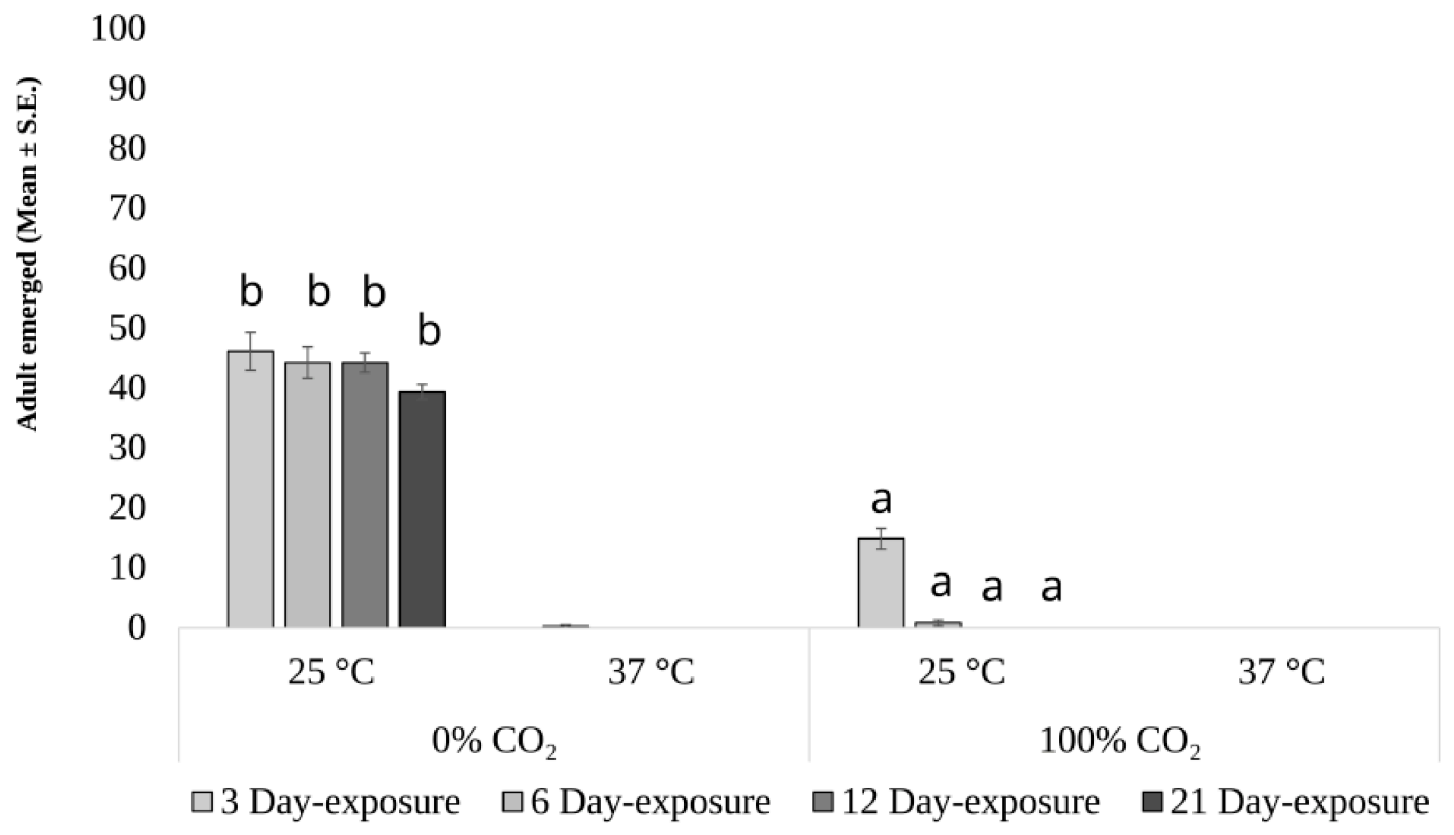
Egg mortality of *S. granarius* expressed by number of adult emergence (Mean ± S.E.) from wheat kernels infested by eggs in atmospheric air or exposed to 100% of CO_2_ at 25 or 37 °C for 3, 6, 12, and 21 days. Different letters indicate significant differences (GLMM post hoc Bonferroni corrected, *p* < 0.05) between CO_2_ doses within the same temperature and exposure day.

For egg-infested wheat in the control, adults emerged ranged from 46.1 ± 3.2 to 39.3 ± 1.3, with no statistically significant variations in relation to the time of exposure (Figure 2). At the same temperature, the 100% CO_2_ treatment induced a significant (*p* < 0.001) reduction in adult emergence after three days of exposure and emergence dropped to almost zero starting from the sixth day of exposure (Figure 2). At 37 °C, adult emergence was close to zero across all exposure times regardless of the presence of CO_2_, demonstrating that thermal stress alone was sufficient to prevent adult emergence from egg-infested wheat. (Figure 2). This total absence of emergence under 37 °C was significantly (*p* < 0.001) lower compared to the values recorded at 25 °C (Figure 2).

**Figure 3:**
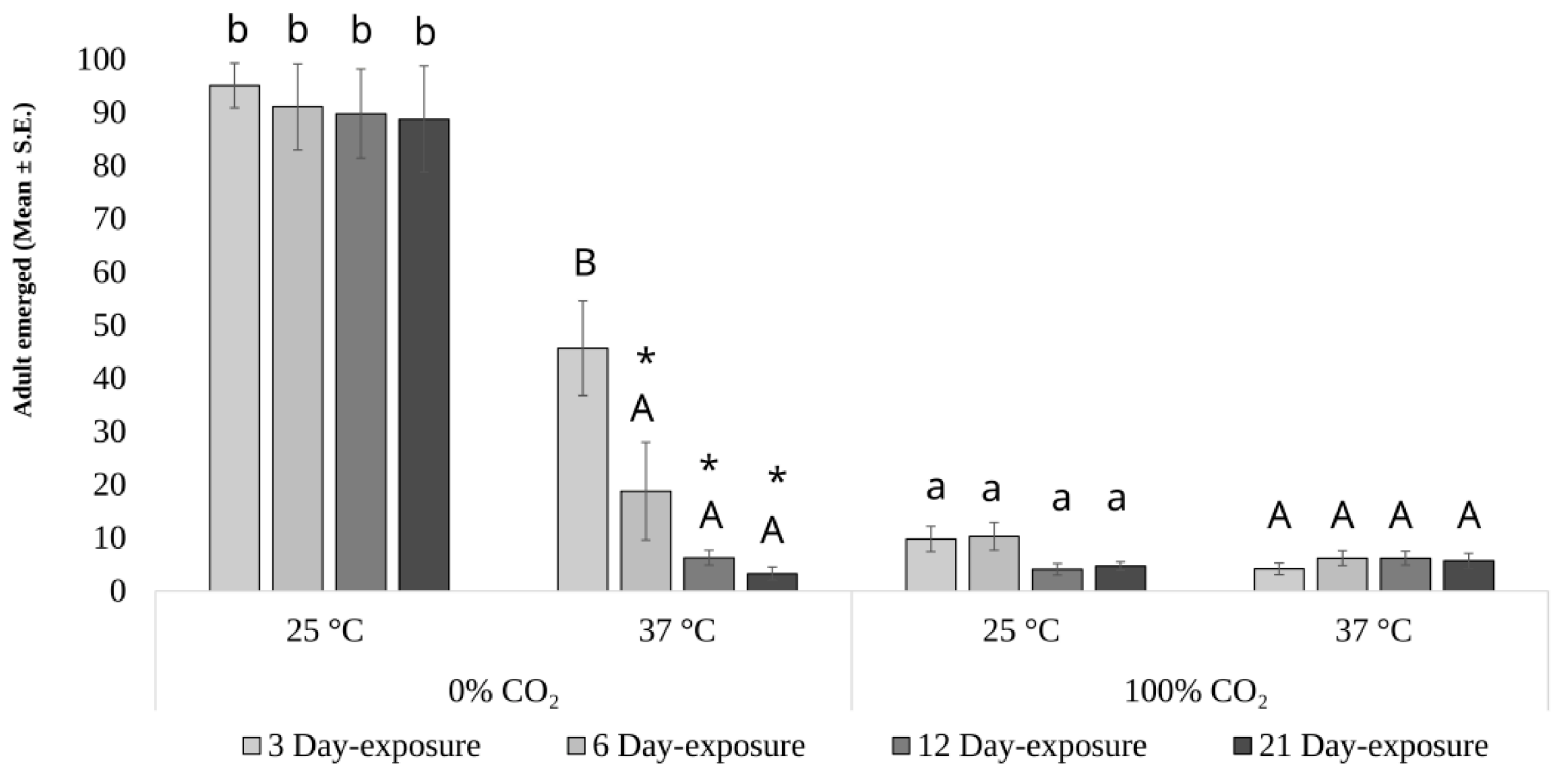
Larvae mortality of *S. granarius* expressed by number of adult emergence (mean ± S.E.) from wheat kernels infested by larvae in atmospheric air or exposed to 100% of CO_2_ at 25 or 37 °C for 3, 6, 12, and 21 days. Different letters indicate significant differences (GLMM post hoc Bonferroni corrected, *p* < 0.05) between CO_2_ doses within the same temperature and exposure day. Asterisks indicate a statistically significant (GLMM post hoc Bonferroni corrected, *p* < 0.05) effect of temperature when comparing the 37 °C thermal condition against the respective 25 °C at the same dose and exposure time.

For larva-infested wheat, the number of emerged adults in the control samples ranged from 101.8 ± 12.1 to 89.8 ± 8.4, with no statistically significant variations over the time (Figure 3). At the same temperature, the application of 100% CO_2_ treatment significantly (*p* < 0.05) reduced the adult emergence, which ranged from 10.3 ± 2.6 to 4.7 ± 0.8 for all exposure durations (Figure 3).

At 37 °C in absence of CO_2_, adult emergence exhibited a time-dependent reduction, ranging from 45.7 ± 8.9 at day 3 to 3.3 ± 1.3 at day 21. In absence of CO_2_ treatment, adult emergence was significantly (*p* < 0.05) lower at 37 °C than at 25 °C from day 6 onward, whereas the difference after three days was not statistically significant (Figure 3).

When larvae were simultaneously exposed to 37 °C and 100% CO_2_, adult emergence ranged from 6.3 ± 1.4 to 3.3 ± 1.2 with no significant variations over time. Under 100% CO_2_ treatment, no significant differences were observed between 25 °C and 37 °C at any of the tested exposure times. Notably, a significant (*p* < 0.05) difference between the two CO_2_ concentrations at 37 °C was detected exclusively at day 3, where the gas treatment induced a further significant reduction in emergence compared to the thermal stress alone.

**Figure 4:**
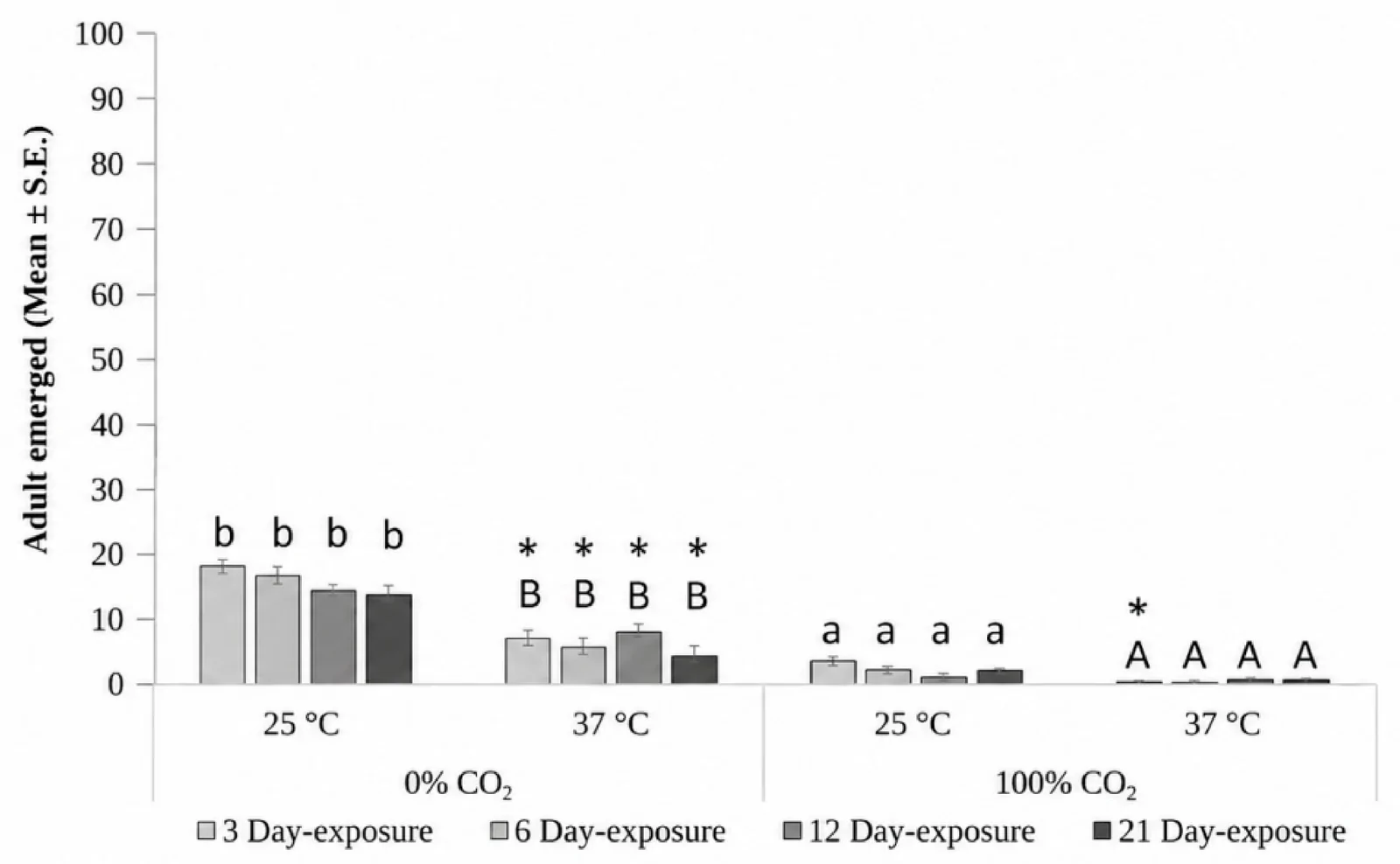
Pupae mortality of *S. granarius* expressed by number of adult emergence (mean ± S.E.) from wheat kernels infested by pupae in atmospheric air or exposed to 100% of CO_2_ at 25 or 37 °C for 3, 6, 12, and 21 days. Different letters indicate significant differences (GLMM post hoc Bonferroni corrected, *p* < 0.05) between CO_2_ doses within the same temperature and exposure day. Asterisks indicate a statistically significant (GLMM post hoc Bonferroni corrected, *p* < 0.05) effect of temperature when comparing the 37 °C thermal condition against the respective 25 °C at the same dose and exposure time.

For pupa-infested wheat, the number of emerged adults in the control ranged from 18.2 ±1.1 to 13.8 ± 1.5 (Figure 4). At the same temperature, the application of 100% CO_2_ treatment significantly (*p* < 0.001) reduced the adult emergence across all exposure durations (Figure 4). At 37 °C in absence of CO_2_, adult emergence ranged from 7.1 ± 1.2 at day 3 to 4.3 ± 1.6 at day 21 and was significantly (*p* < 0.001) lower than that recorded at 25 °C for all days of exposure (Figure 4).

At 37 °C and 100% CO_2_ the adult emergence ranged from 0.3 ± 0.2 to 0.7 ± 0.3 with no significant variations over time. Under 100% CO_2_ treatment, a significant (*p* < 0.05) difference between 25 °C and 37 °C was detected exclusively at day 3, where the gas treatment induced a further significant reduction in emergence compared to the thermal stress alone (Figure 4).

## 4. Discussion

The CO_2_ is registered as a fumigant in several countries [27], however commercial application of high-CO_2_ treatments is often limited by the long exposure periods required and the associated high gas and operational costs [20]. Thus, the research has focused on combining CO_2_ treatment with other physical methods, such as modified temperatures, to achieve a synergistic effect capable of reducing exposure times while maintaining high level of efficacy [20].

The present study evaluated the survival of all *S. granarius* stages (i.e., adults, eggs, larvae, and pupae) exposed to 100% CO_2_ fumigation at 25 or 37 °C for different times of exposure.

According to the results, CO_2_ and temperature, applied individually and in combination, significantly influenced the survival of *S. granarius* for all life stages under examination. However, depending on the development stage of insect, the temperature range, the length of the exposure, and the efficacy of the treatment was not uniform.

Indeed, in the case of adults maintained on wheat at 25 °C and exposed to 100% CO_2_, the total mortality has been achieved after only three days of exposure during which development stops and insects die due to the stresses from hypercarbia, hypoxia, or both, caused by the altered atmospheric concentration of CO_2_ and O_2_ within the storage environment [28]. A temperature of 25 °C is considered optimal for the development and metabolic activity of *S. granarius*; thus, it is possible that the combination of a fully saturating CO_2_ atmosphere and active metabolic demand at this temperature contributed to the rapid lethality observed. A similar trend was reported by Conyers and Bell [18], where 10 to 20% CO_2_, when combined with 20 or 25 °C temperature, caused 95% mortality in *S. granarius* after 28 days of exposure. Another study by Vassilakos et al. [29] found that the application of 70% CO_2_ at 28 ± 2 °C led to 100% mortality after four and six days of the exposure. At 37 °C with 100% of CO_2_ the total mortality was achieved by the third day of exposure, similar to the CO_2_ treatment at 25 °C. This indicates that 100% CO_2_ at 25 °C already exerts maximum lethality on adults within a short exposure window, meaning that the addition of thermal stress of 37 °C does not further enhance treatment efficacy for this specific stage. Conversely, at 37 °C in absence of CO_2_, the total adult mortality was obtained by 12 days of exposure without statistically significant difference due to the presence of 100% CO_2_. This result suggests that the thermal stress at 37 °C in air, sustained over 12 days, was sufficient to achieve 100% of mortality regardless of the atmospheric composition. Previous work has indicated that the upper lethal threshold for *S. granarius* adults lies in the range from 36 to 40 °C which depends on the duration of exposure and relative humidity [30], and the current outcomes are broadly consistent with this range.

Regarding immature stages, eggs proved to be the most sensitive to the combined treatment of CO_2_ and temperature. At 25 °C, the application of 100% CO_2_ progressively reduced adult emergence from egg-infested wheat, reaching complete suppression after 12 days of exposure. At 25 °C in combination with 100% CO_2_, adult emergence from egg-infested wheat decreased, reaching zero after 12 days of exposure. This pattern matches with the sensitivity of eggs to the lack of oxygen. Embryonic development requires active mitochondrial respiration, and anoxic or hypercapnic conditions during critical periods of cell division can cause irreversible developmental arrest [31]. In contrast, exposure to 37 °C resulted in a complete absence of adult emergence across all exposure durations regardless of CO_2_ presence. This demonstrates that thermal stress alone was sufficient to completely suppress adult emergence from egg-infested wheat at 37 °C, independent of the gas composition. This findings suggested that 37 °C is at or near the upper developmental threshold for *S. granarius* eggs, in line with earlier reports that embryonic development in this species is progressively impaired above 32–34 °C [32].

The larval stage exhibited different patterns for the treatments in contrast to eggs, and the primary driving factor for this life stage was CO_2_, with temperature playing a secondary but still significant role. At 25 °C and 100% CO_2_ adult emergence from larva-infested kernels was significantly lower than from the untreated control samples, although not eliminated even after 21 days of exposure. This marked tolerance of *S. granarius* larvae aligns with Lindgren and Vincent [33], who demonstrated that immature stages of *Sitophilus* species require substantially longer exposure periods to reach high mortality levels compared to adults when exposed to carbon dioxide atmospheres. Similarly, Annis and Graver [34] showed that achieving complete control of internal-feeding immature stages in stored grain under modified atmospheres necessitates prolonged exposure regimes, as larval development within the grain matrix delays gas action. This persistent survival over 21 days can be attributed to both physical and physiological barriers. Physically, the compact pericarp and outer layers of the wheat kernel act as a structural barrier to gas penetration, restricting rapid gas exchange and creating a buffered intragranular microenvironment that slows the diffusion of CO_2_ into the internal feeding cavity [35]. Physiologically, endophytic larvae of *S. granarius* exhibit high metabolic plasticity; under severe hypoxia or hypercapnia, they can undergo metabolic rate depression, reducing ATP consumption and entering a quiescence-like state that allows them to survive extended periods of atmospheric stress [22]. This persistent survival of larvae under high-CO_2_ level at standard storage temperature represents a critical finding for practical pest management. Grain weevils are identified as one of the more CO_2_-tolerant stored-product insects compared to species such as *Tribolium castaneum* (Herbst) and *Oryzaephilus surinamensis* (L.), due to their endophytic development [22,36]. Indeed, during the development stage larvae of *S. granarius* are enclosed within grain kernels and the intragranular microenvironment may buffer CO_2_ penetration to some extent, specifically for shorter exposure periods [35]. Consequently, for practical treatment optimization, relying solely on CO_2_ at 25 °C may require either extended holding times. At 37 °C in air the temperature effect was pronounced, with adult emergence declining significantly over the time. Notably, the combination of 37 °C and 100% CO_2_ did not reduce larval emergence as compared to 100% CO_2_ at 25 °C regardless of the exposure time, demonstrating that CO_2_ alone was already suppressing larval survival near to its maximum possible extent. Consistent with these outcomes, Keszthelyi et al. [30] also showed that the treatments with CO_2_ suppressed the adult emergence from *S. granarius* larvae supporting the view that atmospheric stress can limit immature survival and adult emergence.

In the case of pupae, 100% CO_2_ at 25 °C considerably reduced adult emergence by day 3, with a further reduction at days 6 and 21. Similarly, Lindgren and Vincent [33] reported that while pupae are highly susceptible to respiratory disruption, complete mortality across all immature populations often demands extended exposure times due to varying metabolic rates during metamorphosis. Furthermore, Annis and Graver [34] noted that while near-complete reduction in emergence can be achieved rapidly under high-CO_2_ levels, absolute eradication remains challenging without sustained holding periods or thermal synergy. At 37 °C adult emergence in air was significantly lower compared to same treatment at 25 °C regardless of the exposure times. This confirms that elevated temperature alone exerted considerable stress on pupae which halted their development. Emergence was further suppressed when 100% CO_2_ was combined with 37 °C, reaching values near zero at all time points, with no statistically significant differences among exposure durations within this treatment group. Consistent observations were made by Hashem et al. [37], where it was revealed that with the increasing concentration of CO_2_ and exposure periods, the reduction in pupae and adult emergence from larvae of *Sitotroga cerealella* Oliv. was increased across different modified atmosphere conditions. Notably, across any of the applied exposure times, no significant differences were observed between the 100% CO_2_ treatments at 25 °C and 37 °C, suggesting that CO_2_ at 25 °C was already imposing maximum suppressive pressure on pupal development, and the addition of thermal stress at 37 °C did not further enhance efficacy in a statistically meaningful way. These findings are consistent with the known physiological vulnerability of the pupal stage, during which metabolic demands for adult tissue reorganization are high and any disruption to respiratory function through hypercapnia is likely to cause irreversible developmental failure [38].

Finally, in our bioassays, the interaction among gas concentration and temperature was stage-dependent. For highly susceptible stages, such as adults or eggs, one factor alone reached maximum lethality. Conversely, for the more tolerant stages of larvae and pupae, the combined treatment operates through concurrent additive physiological stress.

In conclusion, combining modified atmospheres of CO_2_ with thermal stress represents a promising physical control mean for stored-grain protection, offering a potential residue-free alternative to chemical fumigants. The primary objective of this baseline study was to establish a physiological proof-of-concept by evaluating whether CO_2_, tested at its maximum theoretical potential (100% saturation), could achieve complete control across all life stages of *S. granarius*, both alone and combined with thermal stress. However, the susceptibility pattern of different life stages of *S. granarius* aligns with the clear physiological gradient. The combination of CO_2_ and tested temperatures ensured effective control of adults and eggs, whereas the larvae and pupae results were more tolerant. This highlights that future protocol optimization should be focused specifically on overcoming the tolerance of larvae and pupae. This context will be useful to explore the effect of higher temperatures in combination with CO_2_ in future research. Furthermore, the combined action of gas concentration and temperature often proved more decisive than exposure duration alone, opening up the possibility of investigating shorter treatment windows. These findings may help optimize future application protocols, offering a concrete, sustainable tool to enhance insect control efficiency across the durum wheat value chain. Accordingly, further studies are currently underway to evaluate operationally realistic CO_2_ concentrations more suitable for practical silo applications.

## Figures and Tables

**Table 1:** Results of the Generalized Linear Mixed Model (GLMM) performed with temperature, CO_2_ concentration, and time of exposure as covariates and total number of dead adults or adults emerged from infested wheat as dependent variable. Values represent Wald *F* statistics (*F*), degrees of freedom (*df*), and significance levels (*p*).

Main Effects	Adult Mortality			Adult Emergence								
				Egg-Infested Wheat			Larva-Infested Wheat			Pupa-Infested Wheat		
	*F*	*df*	*p*	*F*	*df*	*p*	*F*	*df*	*p*	*F*	*df*	*p*
Temperature	40.1	1	<0.001	4.3	1	<0.05	97.8	1	<0.001	125.3	1	<0.001
CO_2_ concentration	98.4	1	<0.001	4.9	1	<0.05	227.5	1	<0.001	373.3	1	<0.001
Time	4.2	2	<0.05	3.5	3	<0.05	10.3	3	<0.001	2.8	3	<0.05
Interaction												
Temperature × Concentration × Time	14.6	7	<0.001	3.7	10	<0.001	11.7	10	<0.001	7.6	10	<0.001

## Data Availability

The original contributions presented in this study are included in the article. Further inquiries can be directed to the corresponding authors.
